# Radioactive Quality Evaluation and Cross Validation of Data from the HJ-1A/B Satellites' CCD Sensors

**DOI:** 10.3390/s130708564

**Published:** 2013-07-05

**Authors:** Xin Zhang, Xiang Zhao, Guodong Liu, Qian Kang, Donghai Wu

**Affiliations:** 1 College of Global Change and Earth System Science, State Key Laboratory of Earth Surface Processes and Resource Ecology, Beijing Normal University, Beijing 100875, China; E-Mails: robeson1010@gmail.com (X.Z.); donghai@mail.bnu.edu.cn (D.W.); 2 State Key Laboratory of Remote Sensing Science, Jointly Sponsored by Beijing Normal University and Institute of Remote Sensing Applications, Chinese Academy of Sciences, Beijing 100875, China; 3 School of Geography, Beijing Normal University, Beijing 100875, China; 4 China Center for Resource Satellite Data and Applications, Beijing 100094 China; E-Mails: ji981_31@163.com (G.L.); 13718191905@139.com (Q.K)

**Keywords:** sensor, radioactive quality evaluation, cross validation, HJ-1A/B CCD

## Abstract

Data from multiple sensors are frequently used in Earth science to gain a more complete understanding of spatial information changes. Higher quality and mutual consistency are prerequisites when multiple sensors are jointly used. The HJ-1A/B satellites successfully launched on 6 September 2008. There are four charge-coupled device (CCD) sensors with uniform spatial resolutions and spectral range onboard the HJ-A/B satellites. Whether these data are keeping consistency is a major issue before they are used. This research aims to evaluate the data consistency and radioactive quality from the four CCDs. First, images of urban, desert, lake and ocean are chosen as the objects of evaluation. Second, objective evaluation variables, such as mean, variance and angular second moment, are used to identify image performance. Finally, a cross validation method are used to ensure the correlation of the data from the four HJ-1A/B CCDs and that which is gathered from the moderate resolution imaging spectro-radiometer (MODIS). The results show that the image quality of HJ-1A/B CCDs is stable, and the digital number distribution of CCD data is relatively low. In cross validation with MODIS, the root mean square errors of bands 1, 2 and 3 range from 0.055 to 0.065, and for band 4 it is 0.101. The data from HJ-1A/B CCD have better consistency.

## 1. Introduction

The HJ-1A/B satellites successfully launched on 6 September 2008. HJ is the Chinese abbreviation for “Huan Jing” meaning “environment”. The overall objective is to establish an operational Earth observation system for disaster monitoring and mitigation using remote sensing technology and to improve the efficiency of disaster mitigation and relief. There are two charge-coupled device (CCD) cameras and one hyper-spectral imager (HSI) on-board the HJ-1A satellite. The payload of the HJ-1B satellite also includes two similar CCDs and one infrared scanner (IRS). The four CCD cameras and the HSI operate in the visible and near-infrared regions, while the IRS operates in the shortwave infrared and thermal regions. The four CCD cameras have the same spectrum ranges and spatial resolution [1]. The main sensors of the HJ-1A/B satellites and their characteristics are shown in Table 1.

One of the HJ-1A/B satellites' advantages is that the revisit-time is shortened to less than 48 hours. The CCD data were widely used in wetland [2], crop classification [3], sea ice monitoring [4], drought monitoring [5], land cover classification [6], mapping [7], combined Normalized Difference Vegetation Index(NDVI) use [8] and so on. Based on the contrast of ideal and actual conditions in cross-calibration, eight sources of cross-calibration uncertainty were proposed [9]. Compared with Landsat TM, the red and near-infrared bands of CCD data shows the best quality, including better radiometric resolution, larger image sharpness, information entropy and power spectral summation. Considering the NDVI, the NDVI from HJ CCD data can achieve similar accuracy to the NDVI from Landsat TM data [10]. Joint use of different CCD camera images for quantitative applications requires that they produce consistent and high quality data. Therefore, it is essential to evaluate and cross validate the HJ-1A/B CCD data quantitatively before using these data.

The approaches used for evaluating the quality of remote sensing data are categorized into two different criteria. One is the subjective quality evaluation based on many observers, and the other is the objective quality evaluation based on mathematical calculations [11]. Compared with the subjective methods, quantitative objective image quality testing is easier to implement. Methods such as mean, variance and Angular Second Moment (ASM) are used to analyze the performance of image data. The cross validation method is frequently used in evaluating or calibrating images [12–15], to analyze consistency among many images. In this study, cross validation was done among the HJ-1A/B CCD data. Then, moderate resolution imaging spectro-radiometer (MODIS) data were used to cross validate the data of HJ-1A/B CCD for similar characteristics and spectral coverage.

The calibration consistencies of MODIS, Landsat 7 ETM+, IRS-P6, and EO-1 ALI were evaluated [14–17]. As an extension of the previous study, this paper used calibrated images of the HJ-1A/B CCDs to evaluate radioactive quality and consistency using quantitative evaluation and cross validation methods. These evaluations provide users with a basis for data comparison in a single scene or between images acquired on different dates or by different sensors. The objectives of this study are to evaluate the data consistency and radioactive quality of data from the four CCDs and carry out cross validation among them. This following presentation is organized as follows: Section 2 describes data which were used in this study. Section 3 is focused on methodology and data processing, Section 4 describes results and discussion. Concluding remarks are briefly stated in Section 5.

## 2. Data

Besides the HJ-1A/B CCD data, the MODIS top-of-atmosphere reflectance data were used to validate the radioactive quality of HJ-1A/B CCD data. The MODIS sensor was launched on 18 December 1999 aboard the Terra satellite [18] and has been in operation for over a decade, providing global data that are used to monitor long-term changes in the Earth system [19,20]. The MODIS sensor was designed to image the Earth in 36 spectral bands using detectors located on four focal plane assemblies. MODIS Bands 1–19 and 26, covering the spectral wavelengths from 0.412 to 2.1 μm , are the reflective solar bands, which produce images of the daytime reflected solar radiation. Bands 20–25 and 27–36, with spectral coverage from 3.7 to 14.4 μm , are the thermal emissive bands, which make daytime and nighttime observations of the Earth's thermal emissions. It shows satisfactory spectral performance and stable spatial characterization [21]. The key sensor characteristics of HJ-A/B CCD and MODIS are summarized in Table 2.

**Figure 1.**
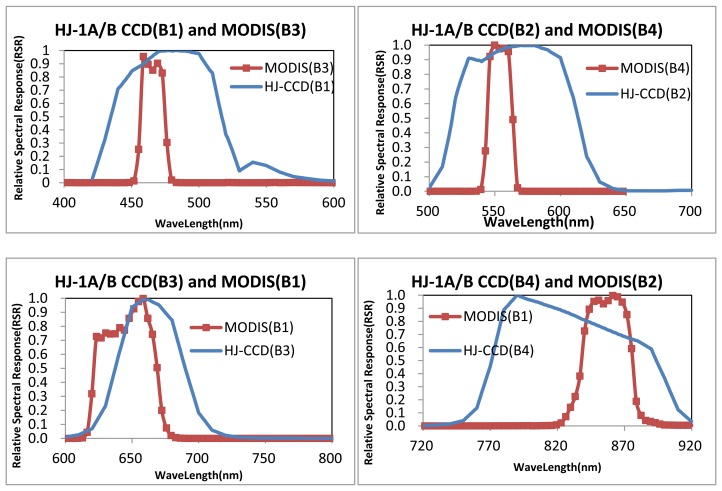
RSR Profiles of HJ-1-A/B CCD and MODIS. The center wavelengths are represented by vertical straight lines in the graphics.

**Figure 2.**
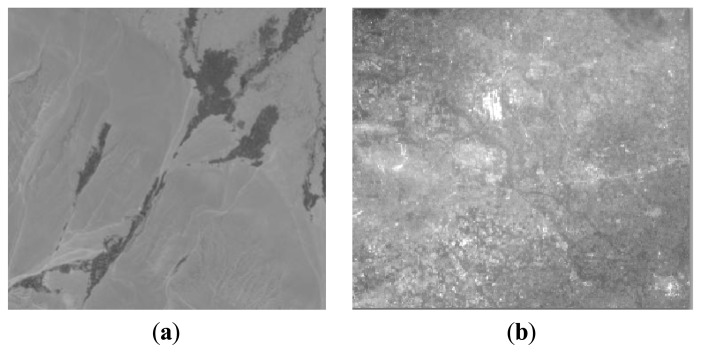
Images used in the radioactive quality evaluation: (a) desert and (b) urban scenarios.

Figure 1 shows the relative spectral response (RSR) profiles of the HJ-1A/B CCD and MODIS sensors measured during prelaunch characterization. Images containing various typical surface objects were used in the radioactive quality evaluation. In the performance evaluation, typical desert (Figure 2a) and urban area images such as those of Beijing (Figure 2b) and Hefei were chosen. In one evaluated area, at least three images were evaluated to avoid stochastic error.

**Figure 3.**
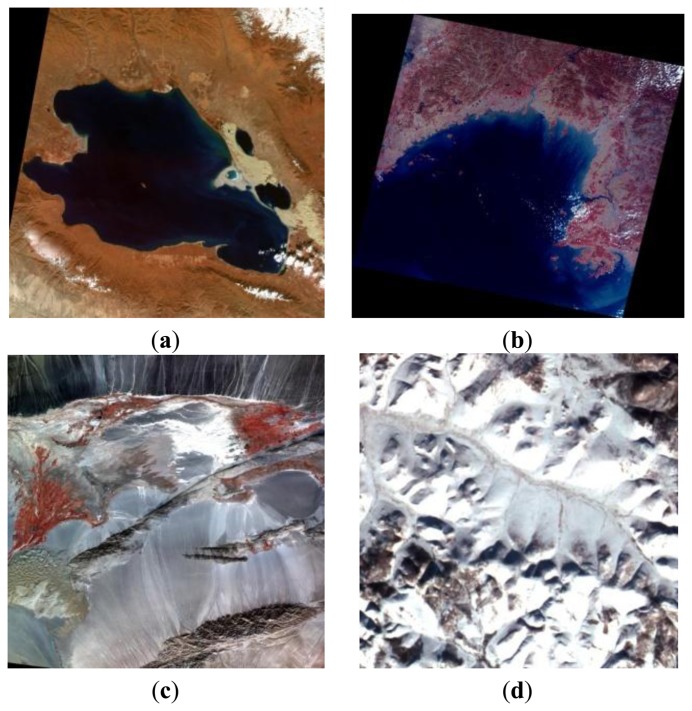
Data used in cross-validation: (a) lake, (b) ocean, (c) desert, and (d) snow.

Images used for cross validation were acquired in the same time and were considered homogeneous targets. Under certain circumstances, lake, snow, desert, and ocean images were chosen (Figure 3) to compare the differences among data from different CCDs. Table 3 presents the image details used in cross validation. For the data acquired within about one month, surface and atmospheric conditions showed little changes. The differences between different CCD dates could be attributed to the results from calibration or payload performances.

**Figure 4.**
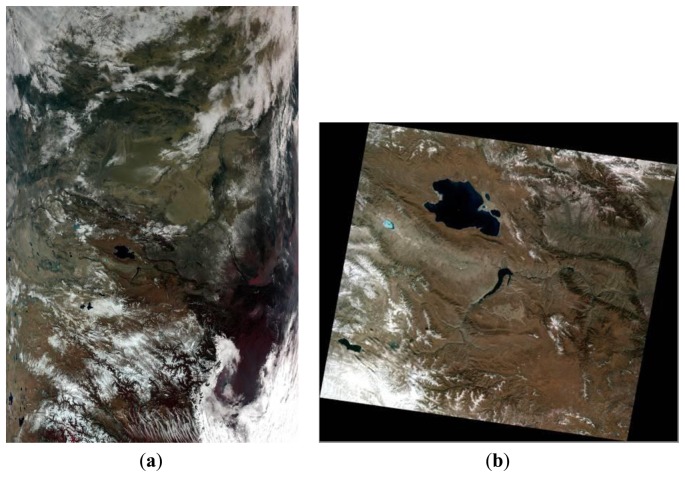
Qinghai Lake images acquired from (a) MODIS and (b) HJ-B CCD2. (Imaging on UTC time 4:00 AM 2 December 2009).

The MODIS Level 1B data set contains calibrated and geolocated at-aperture radiances for 36 bands generated from MODIS Level 1A sensor counts (MOD 01). The radiances are in W/(m^2^·Sr μm). In addition, reflectance may be determined for the solar reflective bands (bands 1−19, 26) through knowledge of the solar irradiance (e.g., determined from MODIS solar-diffuser data, and from the target-illumination geometry). Additional data are provided, including quality flags, error estimates, and calibration data. MODIS and HJ-1B CCD2 data on Qinghai Lake were acquired on the same day (Figure 4). For the data from HJ-1A/B CCD and MODIS acquired at one time, the TOA reflectances were used to perform cross validation.

## 3. Methodology and Data Processing

### 3.1. Radioactive Quality Evaluation Method

Radioactive quality of HJ-1A/B CCD data were evaluated using quantitative indicators [22]. The indicators comprise dynamic range and texture details suggested by Haralick *et al.* [23]. Dynamic range was expressed by mean and variance, and texture details were calculated through ASM, contrast, and edge signal.

Mean is the average value of image data, which can represent image brightness. Variance is the square of the standard deviation. In texture detail, ASM stands for the uniformity of digital number (DN) distribution. It reflects the image gray distribution uniformity and texture roughness. Given that it is the sum of squares of the gray level co-occurrence matrix, it can also be called energy. If the ASM is higher, the texture is richer, and the energy is more intense. ASM is calculated as follows:

(1) $$\text{ASM}=\sum_{i}\sum_{j}{\left\{\frac{p(i,j)}{R}\right\}}^{2}$$

where P(i,j) is the spatial co-occurrence matrix and R is the frequency normalization constant for the selected orientation.

Contrast is a parameter used to evaluate image texture and clarity. When image texture is deeper, the contrast is greater, and the image has better quality. The calculation process used gray level co-occurrence matrix, and its formula is as follows:

(2) $$\text{contrast}=\sum_{n=0}^{L-1}n^{2}\{\sum_{i}\sum_{j}\frac{p(i,j)}{R}\}$$

where |*i−j*|=*n*.

Image edge represents important information about shape characteristic and details. In the template convolution process, convolution computation can be done using the normalized edge operator of two diagonals, 135° (E2) and 45° (E1), and then computing the sum. Edge signal e (x, y) is calculated as:

(3) $$e(x,y)=E_{1}(f(i,j))+E_{2}(f(i,j))=E(f(i,j))$$

### 3.2. Cross Validation Method

The cross validation method consists of two parts: internal cross validation and external cross validation. The internal cross validation method is the validation of data among the HJ-1A/B CCDs, while the external cross validation method is the validation between MODIS and HJ-1A/B CCD data.

Internal cross validation was performed among the data from different HJ-1A/B CCDs. The China Center for Resources Satellite Data and Application (CRESDA) has conducted radioactive calibrated experiments twice. In this study, the calibration coefficients were used to calculate apparent radiance L. Ground reflectance was retrieved from L using the FLAASH [24] atmospheric correction procedure with local atmospheric parameters. Linear relationship among the selected objects of data from different CCDs was analyzed. Then, the slope, correlative coefficients, and root mean square error (RMSE) were calculated [25].

External cross validation was also performed to compare data from HJ-1A/B CCDs and MODIS. Since they were acquired at the same time, they can be compared from the top of atmospheric (TOA) reflectance. The TOA reflectance of the Earth is computed according to the equation:

(4) $$\rho_{\text{TOA}}=\frac{\pi \cdot L\cdot d^{2}}{\text{ESUN}\cdot \cos\left(\theta \right)}$$

where:
ρis the TOA reflectance [unitless],Lis the radiance at the sensor's aperture [W/(m^2^•Sr•μm)],dis the Earth-Sun distance in astronomical units [astronomical units],ESUNis the extraterrestrial solar irradiance [W/(m^2^•μm)]. It is an integrated value over the spectral band of the instrument using the extraterrestrial spectral irradiance spectrum and the RSRs of each band from each instrument. Table 4 shows the ESUN values for HJ-1A/B CCDs.θis the solar zenith angle [degrees].

Prior to their comparison, the HJ-1A/B CCD data were resampled so that both had the same size. The spatial resolutions of HJ-1A/B CCD and MODIS are different; the former is 30 m and the latter is either 250 m or 500 m, depending on the corresponding band. Therefore, the resolution of HJ-1A/B CCD data must be decreased to 250 m or 500 m. Afterwards, linear relationship between the selected objects of data from HJ-1A/B CCD and MODIS was also analyzed. Then, the slope, correlative coefficients, and RMSE were calculated.

*P* value was used to evaluate the correlation. The *p*-value is defined as the probability, under the assumption of no effect or no difference (the null hypothesis), of obtaining a result equal to or more extreme than what was actually observed. In statistical significance testing the *p*-value is the probability of obtaining a test statistic at least as extreme as the one that was actually observed, assuming that the null hypothesis is true [26]. One often “rejects the null hypothesis” when the *p*-value is less than the predetermined significance level which is often 0.05 or 0.01, indicating that the observed result would be highly unlikely under the null hypothesis. Many common statistical tests, such as chi-squared tests or Student's t-test, produce test statistics which can be interpreted using *p*-values.

## 4. Results and Discussion

In order to evaluate the radioactive quality, the texture detail and dynamic range of the histograms of the four HJ-1A/B CCD images, which are covering the regions around Beijing and Hefei, were calculated and analyzed. Table 5 shows the results of this study, which indicate that the balance between each band of HJ-1A/B CCD is good. The DN of the HJ satellite on each band is relatively low on the default gain mode, and the differences among the four bands on one CCD are less than 10. Compared with the distribution of gray level, each band appears narrow and the CCD data are lower than normal, indicating that HJ-1A/B CCD data has a concentrated DN range and a narrower gray-level distribution.

Generally, ASM is used to compare the texture details among different data. Its value indicates whether a given set of data has produced rich texture and smooth image. In addition, the contrast of the image shows the details of the signal and information capacity. Here, the results of the evaluation show that the contrast of each band is generally good, and that the majority contrast are above 30, although there is a large performance difference between each band. In terms of edge signal, the detail signal energy and the edge signal energy of CCD data are more than 4, with some even reaching 180; this indicates good performance. The first band of HJ1-A CCD scores demonstrates the best performance among them.

**Figure 5.**
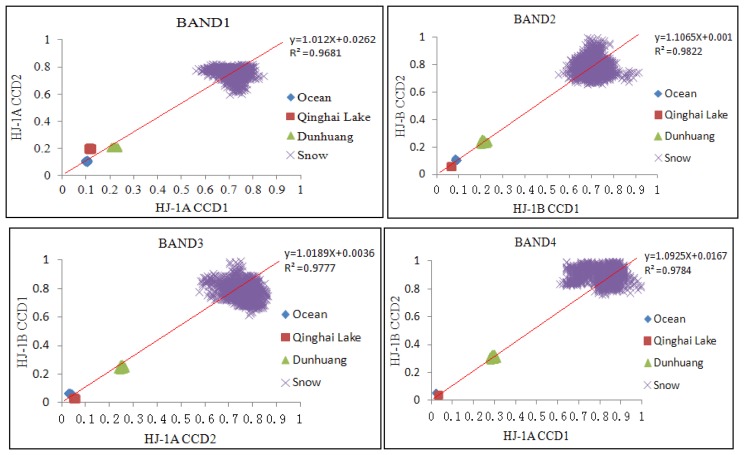
Linear relationship analysis among four HJ CCDs.

A 50 × 50 pixels image was selected on the same area from each scene in HJ satellite CCD data to perform the cross comparison with its ground reflectance. As can be seen from Figure 5, HJ-1A CCD has the best consistency and high reliability under conditions marked by middle-reflectivity features such as Dunhuang.

However, when some features of low-reflectivity such as Qinghai Lake or the ocean are used, bands 1, 3 and 4 showed low consistency. To a certain extent, this reflects that the linearity response of CCD is not so good for the low reflectivity surface features. In addition, for highly reflective surface features such as snow, bands 1 and 4 are less consistent. Table 6 shows the cross validation results among different CCDs, band 4 is less consistent; its RMSE of over 0.1 indicates less reliability of CCD sensors in band 4. Scoring an RMSE between 0.05 and 0.08, bands 1 and 2 are highly correlated and shows high reliability, with the exception of the comparison of band 2 between HJ-1A CCD1 and HJ-1B CCD1. The RMSE of band 3 is between 0.9 and 1, indicating certain reliability of this band.

Table 7 shows the results of cross validation between HJ-1B CCD and MODIS. The P values all below than 0.01 shows that all the correlation are stable. The RMSE of bands 1, 2 and 3 are between 0.055 and 0.065, indicating good correlation. The TOA reflectance of MODIS is slightly less than that of HJ-1A/B CCD. The RMSE of band 4 is more than 0.1, and the slope is 0.6793.

## 5. Summary

An application's most crucial aspects when using many remotely-sensed images is the concern about continuity and consistency. In the case of combined use, data quality on single scene and consistency between images has become a primary requirement; thus, the study of radioactive quality between the sensors is of key interest to the user community. To support these activities, this work focuses on monitoring the quality stability of the HJ-1A/B CCD sensors by using objective quality evaluation and cross validation methods.

In this study, images with typical characteristics and covering the same area, such as urban, desert, ocean and lake images, are chosen to evaluate radioactive quality. The results show that the data quality from HJ-1A/B CCD is stable, and the performances of each band are balanced. Image texture is rich and the images themselves are smooth. The detail energy and other indicators are performing well. From the comparison of HJ-1A/B CCD by using data from different CCDs and MODIS, the relative deviation of the surface reflectance of middle-reflectivity features has reached a very low level, with an RMSE of just within 0.055 to 0.065 when comparing the first three bands. The cross validation related error of band 4 of the CCD camera is 0.101. Furthermore, the results show that data from HJ-1A/B CCD have better consistency compared with those generated from MODIS.

## Figures and Tables

**Table 1.** Spectral and radiometric properties of HJ-1A/B payloads.

**Satellite**	**Payload**	**Band**	**Spectral Range (μm)**	**Spatial resolution (m)**	**Width (km)**
HJ-1A	CCD	B01	0.43–0.52	30	360
		B02	0.52–0.60		
		B03	0.63–0.69		
		B04	0.76–0.90		

	HSI		0.45–0.95	100	50

HJ-1B	CCD	Same parameters as HJ-1A CCDs			

	IRS	B05	0.75–1.10		720
		B06	1.55–1.75	150	
		B07	3.50–3.90		
		B08	10.5–12.5	300	

**Table 2.** Key specifications of HJ-1A/B CCD and MODIS.

**Platform**	**Terra**	**HJ-A/B**
Sensor	MODIS	CCD
Number of bands	36	4
Spatial resolution	250 m, 500 m, 1 km	30 m
Swath	2330 km	700 km
Spectral coverage (μm)	0.4-14	0.43–0.90
Pixel quantization	12 bit	8 bit
Launch date	18 December 1999	6 September 2008
Orbit type	Sun synchronous	Sun synchronous
Equatorial Crossing Time	10:30 AM	10:30 AM
Altitude	705 km	650 km

**Table 3.** CCD data selection display.

**Region**	**Satellite**	**CCD**	**Satellite Orbit (PATH/ROW**)	**Time**	**Sun Elevation**
Lake	A	CCD1	18/82	22 October 2008	39.859
		CCD2	20/72	19 October 2008	42.233
	B	CCD1	18/72	13 October 2008	44.551
		CCD2	17/74	20 October 2008	42.005

Snow	A	CCD1	453/50	25December 2008	13.916
		CCD2	455/51	4 November 2008	21.916
	B	CCD1	453/50	22 November 2008	17.151
		CCD2	454/51	29 October 2008	23.784

Desert	A	CCD1	27/69	16 October 2008	41.48
		CCD2	26/65	4 October 2008	41.691
	B	CCD1	26/65	14 October 2008	39.635
		CCD2	27/65	29 October 2008	33.02

Ocean	A	CCD1	448/67	27 October 2008	36.477
		CCD2	448/66	7 November 2008	31.016
	B	CCD1	449/66	2 October 2008	44.775
		CCD2	448/68	9 October 2008	41.843

**Table 4.** HJ-1A/B CCDs extraterrestrial solar irradiances (units: W/(m^2^·μm)).

**Sensor**	**Band 1**	**Band 2**	**Band 3**	**Band 3**
HJ-1-A CCD1	1914.324	1825.419	1542.664	1073.826
HJ-1-A CCD2	1929.810	1831.144	1549.824	1078.317
HJ-1-B CCD1	1902.188	1833.626	1566.714	1077.085
HJ-1-B CCD2	1922.897	1823.985	1553.201	1074.544

**Table 5.** Radioactive quality evaluation results.

**Region Name**	**Beijing**							
sensor	HJ-1-A CCD1				HJ-1-A CCD2			
band	1	2	3	4	1	2	3	4
DN mean	35.1778	32.5112	37.4015	43.4807	38.5445	32.2765	40.3103	68.7728
variance	19.498	14.5745	12.8044	11.5237	8.7673	14.9149	10.9246	13.5628
ASM	0.0401	0.0238	0.0147	0.069	0.0379	0.0217	0.0134	0.0025
Contrast	878.6485	390.0181	110.7357	1.0586	76.8757	276.809	99.9226	340.0272
edge signal	183.2311	9.1404	42.1443	0.4895	8.1482	45.912	10.7008	21.6412
**Region Name**	**Hefei**							

sensor	HJ-1-B CCD1				HJ-1-B CCD2			
band	1	2	3	4	1	2	3	4
DN mean	32.0065	33.3886	44.2526	40.1942	74.59	26.565	42.7	34.03
variance	6.6385	4.7428	9.4135	9.2383	18.76	6.3177	12.2	11.49
ASM	0.0236	0.0102	0.0046	0.006	0.002	0.0098	0	0.003
Contrast	44.5928	4.3175	36.8409	32.7643	104.5	27.805	41.5	41.51
edge signal	5.8625	4.6524	23.7007	12.6909	72.03	10.697	28.8	33.41

**Table 6.** the cross validation results among different CCDs.

**Band**		**A-CCD1**	**A-CCD1**	**A-CCD1**	**A-CCD2**	**A-CCD2**	**B-CCD1**
		**A-CCD2**	**B-CCD1**	**B-CCD1**	**B-CCD1**	**B-CCD2**	**B-CCD2**
1	Regression	y = 1.012x + 0.0262	y = 1.071x -0.0111	y = 1.098x + 0.002	y = 1.029x -0.0296	y = 1.046x + 0.0172	y = 1.023x + 0.014

	R^2^	0.9681	0.9857	0.9779	0.9629	0.9612	0.9868
	RMSE	0.0676	0.0602	0.0904	0.0554	0.0626	0.0640
	P	<0.01	<0.01	<0.01	<0.01	<0.01	<0.01

2	Regression	y = 1.046x + 0.0172	y = 1.104x -0.0118	y = 1.229x - 0.0136	y = 1.027x -0.0222	y = 1.054x -0.0167	y = 1.106x + 0.001

	R^2^	0.9612	0.978	0.9703	0.9629	0.9526	0.9822
	RMSE	0.0886	0.0818	0.1505	0.0634	0.0996	0.0996

3	Regression	y = 1.052x + 0.0009	y = 1.085x + 0.0011	y = 1.128x + 0.0115	y = 1.019x + 0.0035	y = 1.064x + 0.0131	y = 1.035x + 0.0118

	R^2^	0.9731	0.9759	0.9686	0.9777	0.9779	0.983
	RMSE	0.0915	0.0945	0.1312	0.0746	0.0886	0.0810
	P	<0.01	<0.01	<0.01	<0.01	<0.01	<0.01

4	Regression	y = 0.891x + 0.0113	y = 0.891x + 0.0114	y = 1.092x + 0.0167	y=1.161x −0.0174	y = 1.217x + 0.0054	y = 1.036x + 0.0272

	R^2^	0.9744	0.9827	0.9784	0.981	0.9878	0.9831
	RMSE	0.1066	0.0808	0.1296	0.1290	0.1711	0.1012
	P	<0.01	<0.01	<0.01	<0.01	<0.01	<0.01

**Table 7.** Test results of cross validation.

	**HJ-B1/MODIS-B3**	**HJ-B2/MODIS-B4**	**HJ-B3/MODIS-B1**	**HJ-B4/MODIS-B2**
Regression	y = 0.911x - 0.0321	y = 0.9067x - 0.0324	y = 0.8225x - 0.048	y = 0.6793x - 0.0224
R^2^	0.9069	0.906	0.8518	0.8536
RMSE	0.0569	0.0585	0.0639	0.1010
P	<0.01	<0.01	<0.01	<0.01
